# Beliefs, awareness, use, and factors associated with herbal supplements usage among patients with chronic diseases–A cross-sectional insight from Alkharj, Saudi Arabia

**DOI:** 10.1371/journal.pone.0295116

**Published:** 2024-01-17

**Authors:** Ahmed A. Albassam, Arwa N. Alenzi, Norah K. Alhaqbani, Fatimah K. Alhouty, Ziyad S. Almalki, Ahmed M. Alshehri, Hussain Aldossari, Muhammad Shahid Iqbal

**Affiliations:** 1 Department of Clinical Pharmacy, College of Pharmacy, Prince Sattam Bin Abdulaziz University, Al-Kharj, Saudi Arabia; 2 Pharmaceutical Care Services, King Fahad Specialist Hospital, Buraydah, Saudi Arabia; 3 Pharmacy Department, General Directorate of Medical Services, Ministry of Interior, Al-Kharj, Saudi Arabia; 4 Pharmacy Department, Al-Kharj Military Industries Corporation Hospital, Al-Kharj, Saudi Arabia; 5 Department of Ophthalmology, Al-Kharj Military Industries Corporation Hospital, Al-Kharj, Saudi Arabia; National University of Science and Technology, ZIMBABWE

## Abstract

**Background:**

Herbal supplements (HSs) are used to treat a variety of diseases and ailments. Individuals with chronic diseases are at a higher risk of having adverse events and drug interactions from the use of HSs.

**Aim:**

This study determined the beliefs, awareness, use, and factors associated with HSs usage among patients with chronic diseases in Alkharj, Saudi Arabia.

**Method:**

A cross-sectional study was conducted among patients with chronic diseases between February and June 2019. Face-to-face interviews were conducted at various out-patient clinics in different hospitals. Patients diagnosed with chronic diseases were included in the study. Data were analyzed by descriptive, comparative, and inferential statistics using SAS ver. 9.4.

**Results:**

The study participants were consisted of 533 patients, with mean age 53.6 ±12.9 years. The most prevalent chronic diseases were diabetes mellitus (67.7%), followed by hypertension (54.8%), and hyperlipidemia (53.8%). Among the studied participants, 336 (63%) had used at least one HS, whereby the most commonly used HSs were ginger (74.7%), mint (72%), and cumin (66.7%). Almost 78% of HSs users did not consult any healthcare provider about their use. HSs use varied significantly between female and male participants (*p*<0.05), whereby 61.5% of female participants used HSs in comparison to the male participants (38.5%). Gender (AOR 0.328; 95% CI 0.139–0.772; *p* = 0.0107), number of chronic diseases (AOR 1.585; 95% CI 1.084–2.318; *p* = 0.0312), and hyperlipidemia (AOR 2.818; 95% CI 1.507–5.269; *p =* 0.0.0012) were the pure factors of HSs use among the studied patients.

**Conclusion:**

The results of this study showed that HSs usage was high among patients with chronic diseases in Saudi Arabia. Concurrent usage of HSs with drugs should be well-discussed with healthcare providers to avoid potential adverse events or drug interactions especially among patients with chronic diseases.

## Introduction

Herbal supplements (HSs) are used to treat a variety of diseases like cancer, cardiovascular diseases, diabetes mellitus, anxiety and depression [1–4]. In Saudi Arabia, in 2011, a study reported that individuals spent around $8.2 billion on complementary and alternative medicine (CAM) or HSs usage [1]. Similarly, annual herbal product sales in the United States totaled more than $8.8 billion in 2018 [2]. HSs which are also known as herbs, phytomedicines, botanical or natural medicines are those products whose active ingredients come from plants, parts of plants, plant materials, or that contain a combination of plant-derived substances [3]. These HSs are a vital component of CAM and are sold in a variety of forms, including teas, extracts, capsules, tablets, creams, and powders [4].

Patients suffering from chronic diseases are more likely to use HSs than the general population though general public do use HSs. Alghamdi *et al*., in 2018 found that 68% of the chronic disease sufferers used HSs in Saudi Arabia [5]. Another study done by Alsanad *et al*., in 2018 among Saudi patients with diabetes mellitus reported that the prevalence of CAM users was around 32% where most of the patients used honey, black seeds (*Nigella sativa*), fenugreek (*Trigonella foenum-graecum*), and myrrh (*Commiphora myrrha*) [6]. Another study found that 32% of the chronic liver patients also used HSs [7]. In addition, Al Essa *et al*., in 2019 found that among Saudi patients with neurological disorders, 28% had used HSs [8]. Such widespread use of HSs also increases the risk of adverse events and drug interactions and making it imperative that healthcare providers should better understand the prevalence of HSs use and inquire and counsel their patients about the potential side effects, drug interactions or adverse events of the HSs if they are taken with pharmacological agents.

In Saudi Arabia, few studies have evaluated the prevalence of HSs use and factors affecting their usage among patients with chronic diseases. Majority of the studies have only determined HSs use among the general public in Saudi Arabia [5–7]. In several countries, HSs are licensed as food supplements and thus are not subjected to pre-market safety and efficacy testing [9]. As a matter of fact, HSs are not evaluated by the same standards/measures or procedures as compared to pharmaceutical products or therapeutic agents. Many chronic patients believe and assume that these HSs could cause frequent adverse events and severe drug interactions (herb-drug interactions) when they are consumed with prescribed medicines [10]. Additionally, chronic disease patients using HSs may also have severe adverse events and can experience more frequent and severe drug interactions (herb-drug interactions) than the general public.

In contrast, use of HSs with therapeutic agents (drugs) could be beneficial too if they are used appropriately and precisely. Many patients believe that HSs are safe and harmless by virtue of being natural. However, several clinical evidences have demonstrated that not all HSs are safer and may cause adverse events especially when they are used along with prescribed medications among chronic disease patients [11, 12]. Hence, it is important to determine the exact beliefs, awareness, use, and factors associated with herbal supplements usage among patients with chronic diseases. No previous study has examined the beliefs, awareness, use, and factors associated with HSs usage among patients with chronic diseases in Alkharj. Thus, the present study aimed to determine the beliefs, awareness, use, and factors associated with HSs usage among patients with chronic diseases in Alkharj, Saudi Arabia.

## Methodology

### Study design and study setting

A cross-sectional study was conducted between February and June 2019 among patients with chronic diseases in Alkharj, Saudi Arabia. All aspects of the study protocol including information on participants’ background were strictly confidential and for research purpose only. The Institutional Review Board (IRB) of Prince Sattam bin Abdulaziz University approved the study under PSAU/COM/RC/IRB/P/39 on 28-01-2019.

### Inclusion and exclusion criteria

The study inclusion criteria included adult patients aged ≥ 18, with at least 1 chronic disease and visiting clinics for follow-up and had at least one prescription medication. Participants who met the study inclusion criteria were invited to participate in the study and asked to sign a consent form. Participants were also informed that all data collected shall be kept strictly confidential.

### Sample size

Alkharj is a city with an approx. population of 425300. Study sample size was calculated after adjusting potential nonresponse rate using below formula as described in various studies [13–15],

$$n=\frac{N}{1-Nd^{2}}\;\mathrm{and},$$

$$n_{n}=\frac{n}{1‐d}$$

where n is “required sample size”, N is “total population size”, d is “error margin” (usually 0.05 is an ideal value when “confidence interval/level” is at 95%, n_n_ is “sample size (after consigning nonresponse rate”, n is “earlier calculated sample size”, and d is “nonresponse rate”. After the above calculation, the final sample estimated for this study was 460 whereby we managed to obtain 533. A total of 17 patients denied to participate in the study due to various reasons, i.e., didn’t want to participate, time constrain, and difficulty in recalling the information regarding HSs usage etc.

### Sampling strategy and study site

Convenient sampling technique was used to obtain the required sample size. A face-to-face interview in outpatient clinics at three tertiary hospitals and two primary care facilities were conducted. A total of 157 participants were included from tertiary care hospital 1, 135 from hospital 2, 107 from hospital 3, and 81 from primary care setup 1 and 53 from setup 2. These hospitals and primary care facilities are located in Alkharj, Saudi Arabia.

### Study tool

The study tool was designed after extensive literature review [10, 16–19]. To ascertain the content and face validity, the study tool was validated and pretested by 5 healthcare providers who had experience in treating and managing chronic diseases (2 family medicine physicians, 2 internal medicine specialist, and 1 clinical pharmacist) and by 2 patients who had chronic diseases and had used HSs previously. Cronbach alpha was also measured to obtain the reliability of the research tool. A value of 0.897 was obtained for Cronbach alpha, which ascertained internal consistency (reliability) of the newly developed study tool. The study tool was comprised of 3 sections, where first section asked patients about their demographic and clinical characteristics, medical history, medications they regularly use, and total no. of comorbidities present. The second section asked for information regarding patients’ beliefs and awareness about HSs, such as efficacy, safety, adverse events, cost, and whether they consulted a healthcare provider before using HSs. The third section collected information about the frequency and type of HSs usage among the studied patients. Patients’ disease diagnosis, comorbidities and prescribed medications were retrieved from their medical records. Originally, the study tool was designed in English, and translated to Arabic (local spoken language) by 2 proficient Arabic speakers who were content (subject matter) experts. Then, this version was reverified by reverse translation method by 3 bilingual expert reviewers who were proficient Arabic and English speakers. The study tool was also tested and validated with a pilot study using a group of 30 randomly selected participants. The results of the pilot study were not included in the final analysis. The study tool was revised and all of the inconsistencies were addressed (where needed) after translations and obtaining results of pilot study to make it final and suitable to obtain data from the target population. There was no difficulty observed in reading and understanding the final developed study tool and it was deemed fit to use to obtain the required data.

### Data collection

The developed study tool was distributed by 2 trained and experienced hospital pharmacists. To minimize any potential bias, a standardized data collection process was adopted by the 2 pharmacists during data collection. Both of the pharmacists followed the following steps during data collection, (a) a brief self-introduction, (b) inform patients about study outcomes, (c) explain patients about study benefits to the society, (d) detailed explanation and overall study process (as mentioned on the cover page of the study tool i.e., study information sheet, (e) declaration of anonymous nature of the study, (f) confirmation of patients’ voluntary participation and, (g) obtaining participants’ written consent. Whenever needed, both of the data collecting pharmacists addressed patients’ queries regarding the study and provided more specific information.

### Data analyses

The obtained data were entered and analyzed using SAS version 9.4. To examine the various study variables among study participants (HSs users and non-users), categorical variables were interpreted as frequencies with percentages and continuous variables were presented as mean with standard deviation (SD). Chi-square and/or Fisher’s exact test, were used to evaluate categorical variables between groups. Normally distributed variables were analyzed using Student’s t-test, and non-normally distributed were analyzed using Mann–Whitney U test (Wilcoxon rank-sum). Pure factors (without confounders) of HSs usage were determined using multiple logistic regression model. The measurement of association was taken as the adjusted odds ratio (AOR) with a 95% confidence interval (CI). A *p*-value <0.05 was considered statistically significant.

## Results

### Characteristics of the studied patients

#### Sociodemographic characteristics

A total of 533 patients with chronic diseases participated in the study. Among these 533 patients, 336 (63.1%) were HSs users (used at least one HS) while 197 (36.9%) were non-users of HSs. Patients’ mean age was 53.6 ±12.9 years; the majority were females (56.3%) and resided in an urban area (88.3%). Among the studied patients, 27.0% had elementary school education, followed by 22.9% with no formal education, 22.5% had high school education, and 13.1% had college education. The majority (73.9%) of the studied patients were unemployed. According to the obtained results, patients with age ≤ 50 used more HSs than patients with age > 50 (60.4% vs. 39.6%), respectively. Also, female patients used HSs more than males (63.9% vs. 36.1%), respectively. These results are illustrated in Table 1.

**Table 1:** Sociodemographic characteristics of the studied patients (*n* = 533).

Variable	*n* (%)	HSs usage				*p*-value
		Users		Non-users		
		*n*	(%)	*n*	(%)	
**Patients**	533 (100)	336	63.1	197	36.9	
**Age (Year)**						0.4204
≤ 50	300 (56.3)	203	60.4	97	49.2	
>50	233 (43.7)	133	39.6	100	50.8	
**Gender**						**<0.0001**
Female	300 (56.3)	215	63.9	85	43.2	
Male	233 (43.7)	121	36.1	112	56.8	
**Marital status**						0.7903
Married	444 (83.3)	281	83.6	163	82.7	
Not married	89 (16.7)	55	16.4	34	17.3	
**Residence**						0.2731
Urban	471(88.4)	293	87.2	178	90.4	
Rural	62 (11.6)	43	12.8	19	9.6	
**Education level**						0.3168
Illiterate/No formal education	122 (22.9)	68	20.2	54	27.4	
Elementary school education	144 (27.0)	96	28.6	48	24.4	
Intermediate school education	77 (14.4)	48	14.3	5.4	14.7	
High school education	120 (22.5)	81	24.1	39	19.8	
College education	70 (13.1)	43	12.8	27	13.7	
**Employment status**						0.7398
Employed	139 (26.1)	86	25.6	53	26.9	
Not employed	394 (73.9)	250	74.4	144	73.1	

#### Clinical characteristics

Among the total studied 533 patients, 242 patients had ≤ 2, 216 patients had 3–4, while 75 of the patients had > 4 chronic diseases. A total of 361 patients had diabetes mellitus, whereby 230 (68.4%) were HSs users, and 131 (66.5%) were non-users. A total of 292 patients had hypertension, whereby 180 (53.6%) were HSs users, and 112 (56.8%) were non-users of HSs. Similarly, around 287 patients had hyperlipidemia, whereby 196 (58.3%) were HSs users, and 91 (46.2%) were non-users of HSs. Patients with ≤ 2 chronic conditions used more HSs than patients with > 4 chronic conditions (40.2% vs. 17.0%), respectively. Patients with hyperlipidemia used more HSs than patients without hyperlipemia (41.7% vs. 58.3%), respectively. However, patients with asthma and heart disease used less HSs than patients without asthma and heart disease. The details of the HSs users and non-users vs. presence of chronic diseases are illustrated in Table 2.

**Table 2:** Clinical characteristics of the studied patients (*n* = 533).

Variable	*n*	HSs usage				*p*-value
		Users		Non-users		
		***n***	**(%)**	***n***	**(%)**	
**Number of chronic diseases**						**0.0024**
≤ 2	242	135	40.2	107	54.3	
3–4	216	144	42.8	72	36.6	
> 4	75	57	17.0	18	9.1	
**Comorbidities**						
**Diabetes mellitus**						0.6412
No	172	106	31.6	66	33.5	
Yes	361	230	68.4	131	66.5	
**Hypertension**						0.4625
No	241	156	46.4	85	43.2	
Yes	292	180	53.6	112	56.8	
**Hyperlipidemia**						**0.0066**
No	246	140	41.7	106	53.8	
Yes	287	196	58.3	91	46.2	
**Asthma**						**0.0346**
No	485	299	88.9	186	94.4	
Yes	48	37	11.1	11	5.6	
**Arthritis**						0.4054
No	494	309	91.9	185	93.9	
Yes	39	27	8.1	12	9.1	
**Heart disease**						**0.0341**
No	453	294	87.5	159	80.7	
Yes	80	42	12.5	38	19.3	
**Kidney disease**						0.4477
No	513	325	96.7	188	95.4	
Yes	20	11	3.27	9	4.6	
**Cancer**						0.8531
No	527	332	98.8	195	98.9	
Yes	6	4	1.2	2	1.1	
**Depression**						0.1578
No	511	319	94.9	192	97.5	
Yes	22	17	5.1	5	2.5	
**Neurological disease**						0.6886
No	506	318	94.6	188	95.4	
Yes	27	18	5.4	9	4.57	
**Thyroid disease**						0.1499
No	412	253	75.3	159	80.7	
Yes	121	83	24.7	38	19.3	
**Gastric peptic ulcer**						0.1780
No	505	315	93.7	190	96.5	
Yes	28	21	6.3	7	3.5	
**Others**						**<0.0001**
No	382	218	64.9	164	83.3	
Yes	151	118	35.1	33	16.7	

#### Chronic diseases among HSs users

The most prevalent chronic diseases among the studied cohort of the patients were diabetes mellitus (67.7%), followed by hypertension (54.8%), and hyperlipidemia (53.8%). Least three prevalent chronic diseases were asthma (9%), rheumatoid arthritis (7.3%), and peptic ulcer (5.25%). Table 3 shows detailed presence of chronic diseases among the studied patients.

**Table 3:** Chronic diseases among the studied patients.

Disease	*n*	%
Diabetes mellitus	361	67.7
Hypertension	292	54.8
Hyperlipidemia	287	53.8
Thyroid disorders	121	22.7
Heart disease	80	15.0
Asthma	48	9.0
Rheumatoid arthritis	39	7.3
Peptic ulcer	28	5.25

#### Patients’ beliefs and awareness about HSs usage

Most of the patients believed that HSs had strong healing power (63.7%), and they could be used anytime (59.8%). While comparing to therapeutic medications, a majority of HSs users believed that HSs were more easily available than medications (69.3%), cost-effective (86.6%), and had fewer adverse events (79.5%). Conversely, 56.5% did not believe that HSs are better than medication, and 70.5% believed that HSs have the same therapeutic effect as medications. Around 53.3% of the patients believed that no clinical advice needed from the healthcare providers to use HSs. These results are presented in Table 4.

**Table 4:** Patients’ beliefs and awareness about HSs usage.

Questions	Yes n (%)	No n (%)
HSs have strong healing power	214 (63.7)	122 (36.3)
HSs can be used anytime	201 (59.8)	135 (40.2)
HSs are easily available than medications	233 (69.3)	103 (30.7)
HSs are cost-effective than medications	291 (86.6)	45 (13.4)
HSs are timesaving and effortless in buying	251 (74.7)	85 (25.3)
HSs have no adverse events	267 (79.5)	69 (20.5)
HSs are better than medications	146 (43.5)	190 (56.5)
HSs have same therapeutic effect as medications	99 (29.5)	237(70.5)
I do not need to get any advice from healthcare provider to use HSs	179 (53.3)	157 (46.7)

#### Types of HSs and their use among patients

**Fig 1:**
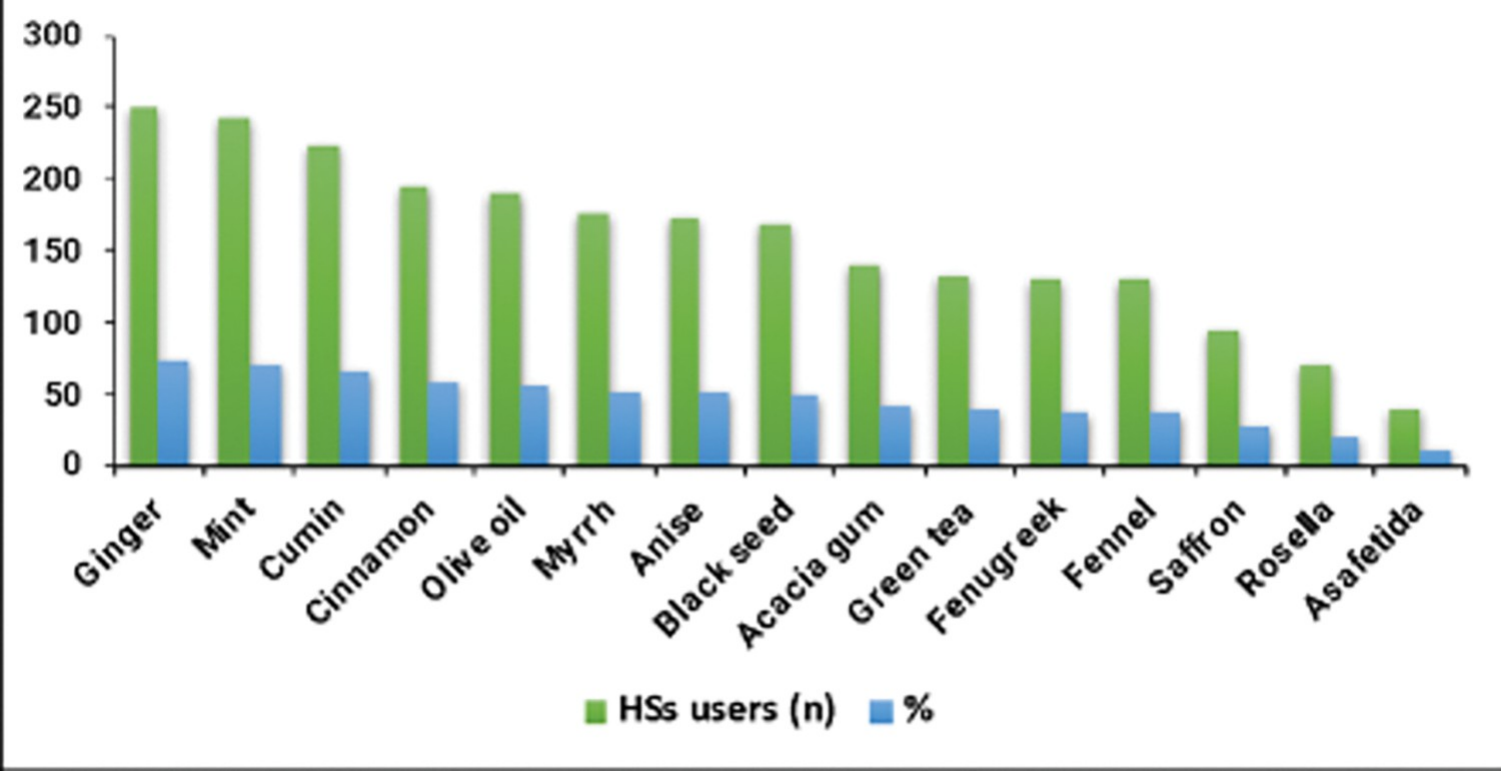
Main HSs types and frequency and percentage of the HSs users.

Among the total 336 (63%) HSs users, the most commonly used HS was ginger (74.7%), followed by mint (72.0%), and cumin (66.7%). Fig 1 shows most commonly used HSs among the studied patients in detail.

#### Factors of HSs usage

Univariate analysis showed a statistically significant relationship in various studied variables like gender (*p* < 0.0001), number of chronic diseases (*p* = 0.0024), hyperlipidemia (*p* = 0.0066), asthma (*p* = 0.0346), heart disease (*p* = 0.0341), and others (misc.) diseases (*p* < 0.0001) among the HSs users. The results of multivariate logistic regression analysis indicated that among all the variables which had statistically significant association with HSs use in univariate analysis, only gender (AOR 0.328; 95% CI 0.139–0.772; *p* = 0.0107), number of chronic diseases; 3–4 chronic conditions (AOR 1.585; 95% CI 1.084–2.318; *p* = 0.0312), >4 chronic diseases (AOR 2.510; 95% CI 1.395–4.517; *p* = 0.0163) and hyperlipidemia (AOR 2.818; 95% CI 1.507–5.269; *p* = 0. 0.0012), had a statistically significant association with HSs use. These results are presented in Table 5.

**Table 5:** Factors of HSs usage among the study participants.

Variable	AOR	95% CI		*p*-value
		Lower	Upper	
**Gender**				
Female	1			
Male	0.328	0.139	0.772	**0.0107**
**Number of chronic diseases**				
≤ 2	1			
3–4	1.585	1.084	2.318	**0.0312**
> 4	2.510	1.395	4.517	**0.0163**
**Comorbidities**				
**Hyperlipidemia**				
No	1			
Yes	2.818	1.507	5.269	**0.0012**
**Asthma**				
No	1			
Yes	1.441	0.474	4.388	0.5197
**Heart disease**				
No	1			
Yes	0.638	0.273	1.490	0.2993
**Others**				
No	1			
Yes	0.417	0.068	2.546	0.3435

Abbreviations: AOR = Adjusted Odds Ratio; CI = Confidence Interval

## Discussion

Patients commonly use HSs to promote their overall health and treat or manage various diseases. Several studies have reported a high prevalence of using HSs among patients with chronic diseases in different countries [20–22]. In this study, it was found that 63% of the patients with chronic diseases used a least one HS. Similar findings were reported in Saudi Arabia (different region) and in Jordan, where 68% and 57.4% of HSs usage was presented, respectively [5, 23]. Conversely, the percentage of the HSs usage was less in another study which was conducted among geriatric Turkish population i.e., 30% [24]. Thus, it was of significant importance to determine the beliefs, use, awareness, and associated factors of HSs use among patients with chronic diseases to avoid adverse events and potential drug interactions.

It was observed that the main reason behind using HSs in the current cohort of patients was to treat chronic diseases (58%), whereas 30.4% of the participants mentioned that they used HSs daily. Likewise, a study among Jordanian patients using herbs declared that curing disease was the leading cause of using various herbs [25]. It is evident in literature that patients with chronic diseases often use HSs along with their prescribed medications. This has to be noticed by healthcare providers to help patients managing any adverse effects occurring because of their concurrent use of HSs with medicines [10, 22–25]. Out of total 300 female patients, 63.9% were HSs users while 43.2% were non-users. Similarly, out of total 287 hyperlipidemic patients 196 (58.3%) were HSs users, and 91 (46.2%) were non-users. Patients with hyperlipidemia used more HSs than patients without hyperlipemia (58.3% vs. 46.2%). Among 48 asthmatic patients, 11.1% were HSs users while 5.6% were non-users. Similarly, among 80 heart disease patients, 12.5% were HSs users while 19.3% were non-users. However, patients with asthma and heart disease used less HSs than patients without asthma and heart disease.

Precise knowledge about use, safety, efficacy and possible adverse events which may result due to inappropriate usage of HSs is important for patients especially if they are suffered from chronic diseases. Several patients consume HSs just because they think that HSs are safe and effective since they are obtained from natural resources. On the other hand, due to the scarcity of the scientific data regarding the safety and efficacy of HSs, patients suffering from chronic diseases often prefer them over therapeutic regimens without knowing that there are numerous active ingredients that which may be present in their HSs and possibly might affect or even alter the desired therapeutic effects of the pharmacologic formulations or medicines [3, 26]. In our study results, it was observed that around 73% of the patients were not aware that HSs were safe or not. Our study results were similar to various other studies in this context as several studies reported the same outcome. A study was done by Kamel *et al*., in 2017 on diabetes mellitus patients in Saudi Arabia confirmed that 54.2% of the study participants did not experience any side effects while they were on HSs [27]. Another study also evidenced similar results where the majority of the studied patients who were suffering from various chronic conditions believed that herbs were safer to use [28]. Patients generally and patients with chronic disease especially have shown in several studies to have story beliefs on HS benefit and safety and therefore, it’s important to improve their knowledge about by holding and promoting awareness camping [26–28].

Healthcare providers can play a crucial role in informing patients about the proper use of HSs and in preventing potential adverse events among them. During patient counselling, HSs type and usage pattern should be reviewed and discussed with patients to minimize possible side effects and adverse consequences. However, the majority of the patients in our study neither consult their healthcare providers before taking HSs (>78%) nor discuss their usage pattern with them (>81.1%). On the other hand, more than 89% of the studied patients stated that their healthcare providers also did not ask or provide them any recommendations about any HSs use or avoidance. In agreement with our findings, 62% of diabetes patients in another study also did not inform their healthcare providers about any herbs they used in conjunction with their medicine, and 64% reported that their healthcare providers did not inquire about their HSs usage too [27]. In another study from the southern part of Saudi Arabia, 88.7% of patients with chronic illnesses reported that they did not discuss their use of herbal remedies with their doctors, and more than 90% said that their doctors also did not inquire about their use of herbal remedies [5]. Similar findings were reported from another country, where more than 77% of the patients with chronic conditions reported that they had not talked to their doctor about using herbal remedies [25]. Our study reports are also in accordance to another study conducted in Turkey, where 42% of elderly patients failed to disclose their use of herbal products to their doctors, but over 40% did so when questioned [24]. Perhaps, one of the most effective methods for addressing this problem could be that healthcare providers should inquire from their patients about their usage of HSs to avoid potential herbal interactions or possible adverse events.

The majority of the patients do believe that HSs have strong healing effects and they do not pose any harm since they are of natural origin or they are natural or herbal products. Most of the participants in our study believed the same that HSs they used, had strong healing power (63.7%) and did not need any certified healthcare provider’s permission (53.3%). A similar finding was also observed in another study where 81.2% of the study participants believed that herbal products are harmless [29]. In our study finding, it was also noted that patients who utilized HSs felt that HSs are more easily available than pharmaceutical products or medicines (69.3%), and had fewer side effects (86.6%). Interestingly, 43.5% of the studied patients also believed that HSs are better than therapeutic medicines, while 29.5% believed that HSs could have the same effectiveness as pharmacological regimens.

The results of this study reported that ginger, mint (peppermint), and cumin were the most frequent HSs used among the studied patients suffering from chronic diseases. Ginger has been traditionally used to treat various ailments, including nausea, motion sickness, vomiting, and arthritis [30]. Mint has a long history of being used as a digestive aid due to its antispasmodic properties. Mint oil has also been used from decades to treat irritable bowel syndrome and non-ulcer dyspepsia. Moreover, many studies reported that people often inhale steam of mint leaves to treat cough and upper-respiratory tract problem [31, 32]. On the other hand, cumin has also been used to treat colic, diarrhea, bloating, and bowel spasms [33]. Several studies have reported a similar pattern of use of various HSs like ginger, mint, and cumin [34, 35]. It is recommended that healthcare providers gain more medical insight about HS by starting with the commonly consumed HS to facilitate their discussion with their patients.

Various factors (after adjusting confounders) were found to be associated with the use of HSs among the studied cohort of patients suffering from chronic diseases. First pure factor was gender, which showed a statistically significant association (*p* = 0.0107) regarding the use of HSs among the studied patients, whereby female patients were found to use more HSs than the male patients with the odds of 0.328. Second factor (without confounders) was the presence of chronic diseases among the studied patients. The usage of HSs was significantly increased among the patients with multiple chronic diseases. Furthermore, HSs use was significantly observed more among patients with hyperlipemia, than heart disease, and asthma patients, with the odds of 2.818. Previous studies have also reported similar findings that females tend to use more HSs than males [36, 37], and patients with chronic diseases are among the substantial and frequent users of HSs [38]. More attention is needed for these specific populations to avoid any unwanted adverse effects.

### Strength and limitations of the study

#### Strength of the study

The findings of this study give baseline data that may be used to develop various training programs and design interventions for healthcare providers for better counselling of the HSs users to avoid unwanted adverse events and drug interactions.

#### Limitations of the study

There are a few limitations of this study. First, there is a risk of recall bias as in many cross-sectional studies, participants’ responses cannot be independently verified. Second, because the study was done in one specific part of Saudi Arabia, it may not be representative of all governorates of the country. However, the majority of the HSs included in this study are common in Saudi Arabian population. Third, because this was a cross-sectional study, no causal implications can be inferred. Fourth, this study was conducted using a convenient sampling technique that may limit the external validity of the obtained results as replicating results is a major concern encounter during convenient sampling method. Fifth, researcher bias may also occur during compilation of obtained data.

## Conclusion

The current study determined beliefs, awareness, use, and factors associated with herbal supplements usage among patients with chronic diseases in Saudi Arabia. More than half of the studied patients had used at least one HS in this study. The majority of patients did not tell their healthcare providers about their HSs use, and the majority of healthcare providers also did not inquire about it. Gender, number of chronic diseases, and hyperlipidemia were the pure factors of HSs use among the studied patients. Patients should be urged to disclose their usage of HSs to the healthcare providers to have an optimized healthcare.
