# Correction: Beliefs, awareness, use, and factors associated with herbal supplements usage among patients with chronic diseases–A cross-sectional insight from Alkharj, Saudi Arabia

**DOI:** 10.1371/journal.pone.0308595

**Published:** 2024-08-06

**Authors:** Ahmed A. Albassam, Arwa N. Alenzi, Norah K. Alhaqbani, Fatimah K. Alhouty, Ziyad S. Almalki, Ahmed M. Alshehri, Hussain Aldossari, Muhammad Shahid Iqbal

After publication of this article [1], concerns were raised about the relationship of this work to a previously published article [2] that was cited and discussed in [1] as Reference 10.

The two studies address different objectives but are part of the same larger project. The objective of [2] was to examine the potential herbal drug interactions among patients with chronic diseases, while the objective of [1] was to study the prevalence of using herbal supplements among patients with chronic diseases and the factors associated with herbal supplement usage [1].

The population sizes and study dates reported in the two articles differ because the study in [2] used only herbal supplement users’ data (336 patients). The authors prolonged the study period beyond the planned end date but did not receive further responses between June and August 2019.

The same herbal supplement user population was used in both studies [1, 2], and so data describing this group were duplicated in the following figures and tables:

- Table 1 of [1] and [2]
- Table 2 of [1] and Tables 2 and 5 of [2]
- Fig 1 of [1] and Table 4 of [2]

PLOS reviewed this matter and concluded that the two articles [1, 2] make distinct contributions to the published literature, although the relationship between the articles and the reuse of data and participants ought to have been disclosed transparently in [1]. The authors apologize for this omission.

In addition, Reference 10 was erroneously cited in the Introduction of [1] to support the following:

“As a matter of fact, HSs are not evaluated by the same standards/measures or procedures as compared to pharmaceutical products or therapeutic agents, Many chronic patients believe and assume that these HSs could cause frequent adverse events and severe drug interactions (herb-drug interactions) when they are consumed with prescribed medicines [10].”

These sentences/citations are hereby updated to:

“As a matter of fact, HSs are not evaluated by the same standards/measures or procedures as compared to pharmaceutical products or therapeutic agents [3]. Many chronic patients believe and assume that these HSs could cause frequent adverse events and severe drug interactions (herb-drug interactions) when they are consumed with prescribed medicines [4–5].”

The Data Availability statement for [1] was incorrect. The individual-level underlying data for [1] are provided here in S1 File.

## Supporting information

S1 File De-identified data for all participants of article [1]. (XLSX)
